# Development and Validation of a Novel Real-time Assay for the Detection and Quantification of *Vibrio cholerae*

**DOI:** 10.3389/fpubh.2017.00109

**Published:** 2017-05-19

**Authors:** Ridwan Bin Rashid, Jannatul Ferdous, Suhella Tulsiani, Peter Kjaer Mackie Jensen, Anowara Begum

**Affiliations:** ^1^Department of Microbiology, University of Dhaka, Dhaka, Bangladesh; ^2^Section for Global Health, Institute of Public Health, University of Copenhagen, Copenhagen, Denmark; ^3^Copenhagen Centre for Disaster Research, Copenhagen, Denmark

**Keywords:** *Vibrio cholerae*, OmpW, *C*_T_ value, sensitivity and specificity, gene copy number, real-time PCR

## Abstract

*Vibrio cholerae* O1 and O139 has been known for its ability to cause epidemics. These strains produce cholera toxin which is the main cause of secretory diarrhea. *V. cholerae* non-O1 and non-O139 strains are also capable of causing gastroenteritis as well as septicemia and peritonitis. It has been proven that virulence factors such as T6SS, *hapA, rtxA*, and *hlyA* are present in almost all *V. cholerae* strains. It is imperative that viable but non-culturable cells of *V. cholerae* are also detected since they are also known to cause diarrhea. Thus, the aim of this study was to develop an assay that detects all *V. cholerae* regardless of their serotype, culturable state, and virulence genes present, by targeting the species specific conserved *ompW* sequence. The developed assay meets these goals with 100% specificity and is capable of detecting as low as 5.46 copy number of *V. cholerae*. Detection is rapid since neither lengthy incubation period nor electrophoresis is required. The assay had excellent repeatability (CV%: 0.24–1.32) and remarkable reproducibility (CV%: 1.08–3.7). Amplification efficiencies in the 89–100% range were observed. The assay is more economical than Taqman-based multiplex real-time PCR assays. Compared to other real-time assays, the *ompW* assay is specific and sensitive, has better repeatability and reproducibility, and is more economical.

## Introduction

*Vibrio cholerae* is a Gram-negative, comma shaped facultative pathogen responsible for causing cholera. The global incidence of cholera was about 2.8 million cases per year, with 91,000 deaths (1). *V. cholerae* O1 has been the etiological agent for several cholera epidemics. The serogroup O139 was responsible for cholera outbreaks in India and other countries in Asia during 1992 (2) and was also isolated during the outbreak in November 2000 in India (3) and March–April 2002 in Bangladesh (4).

*Vibrio cholerae* O1 and O139 serogroups express toxin coregulated pilus which confers the bacteria the ability to colonize the intestine while the cholera toxin is associated with secretory diarrhea (5). Depending on severity, the infectious dose for *V. cholerae* varies from 10^6^ to 10^11^ cells (6).

Toxigenic and non-toxigenic non O1, non-O139 have been documented as incriminating in several outbreaks in developing countries (7–10). In non-CT-producing vibrios, virulence factors such as type 3 secretion systems, hemolysin (HlyA), repeat in toxin (RTX), and heat-stable enterotoxin have major roles in causing infections (11). Hasan et al. (10) reported 98% *V. cholerae* strains carried hemagglutinin protease *hap* (98%) irrespective of their source, i.e., clinical or environmental. Other virulence factors present are T6SS (94–99%), *rtxA* (96%), *toxR* (87%), and *hlyA* (83%), and all these virulence factors might be responsible for diarrhea caused by non-toxigenic non-O1/non-O139 variants.

*Vibrio cholerae* in the viable but non-culturable (VBNC) state can express virulence factors required to produce infection (12). The VBNC cells have the capacity to revert to the culturable state and colonize the intestine (13) the mechanism of which is largely unknown (14). These organisms may go undetected if conventional culture based methods are used (15). Conventional identification of *V. cholera*, which may be done by biochemical tests, is time consuming and laborious. Available commercial biochemical identification systems, such as dipstick test used for the detection of O1 and O139 strains, are not always accurate (16). *V. cholerae* has been shown to possess similar biochemical properties with other species in the Genus *Vibrio* and *Aeromonas*, hence complicating an accurate identification (17).

Compared to conventional PCR, real-time PCR is less labor intensive, more safe, and rapid due to the elimination of gel electrophoresis. It has greater sensitivity and can detect minute amounts of target amplicons that might be missed by the conventional PCR. Real-time PCR can directly target genomic DNA and thus eliminate extensive incubation periods (18). Furthermore, VBNC cells can be detected which might be missed by culture-based methods. The *ompW* sequence is highly conserved among *V. cholerae* species belonging to different biotypes and/or serogroups (17). Hence, the *ompW* gene could be used as a target for species-specific detection, identification, and quantification.

A number of assays exist for the detection of *V. cholera*e (19–24) but many of these assays lack empirical data for reproducibility and repeatability. Some of these assays have not been validated in terms of detecting non-specific products that might accompany the amplification reaction. Furthermore, a number of assays are based on virulence factors that might not be present in certain strains and might yield false negative results.

The aim of this study was to develop an assay that detects and quantifies both O1/O139 and non O1/O139 disease causing strains of *Vibrio* spp. In addition, the assay would be able to quantify VBNC cells that cannot be detected or quantified by conventional methods.

## Materials and Methods

### Assay Controls and Growth Conditions

A total of 28 bacterial strains were used as assay controls. *V. cholerae* strains were grown in alkaline peptone water for enrichment, and all other strains were grown in nutrient broth for 24 h in 37°C. Genomic DNA from overnight cultures controls were extracted and purified according to the manufacturer’s instructions by QIAamp DNA mini kit (Qiagen, Hilden, Germany).

### Sample Preparation and Spiking

Four different types of samples were taken for experiment: (i) drinking water, (iii) pond water, (ii) boiled rice, and (iii) shrimp. Rice sample were prepared by homogenization of 25 g of boiled rice with 225 μL of phosphate-buffered saline (1 L distilled H_2_O, 10 g L^−1^ NaCl, 0.25 g L^−1^ KCl g L^−1^, 1.8 g L^−1^ Na_2_HPO_4_, 0.3 g L^−1^ KH_2_PO_4_; pH 7.4) in a Stomacher Lab Blender (Seward Stomacher^®^ 80, Lab Biomaster, UK). Shrimp sample was also prepared by following the same procedure for rice. All of the samples were spiked with different concentrations of *V. cholerae* CT^+^ O139, *V. cholerae* CT^+^ O1, and *V. cholerae* CT^−^ non-O1/non-O139. Prior to the evaluation of this assay for these environmental samples, absence of *V. cholerae* was confirmed by qPCR. DNA extraction was conducted using QiaAmp^®^ DNA mini kit (Qiagen GmbH, Hilden, Germany) according to the manufacturer’s instruction.

### PCR Primer Design

The Outer Membrane Protein W-OMPW Sequence of eight reference strains (Table 1) was downloaded from the NCBI database. The primer design was accomplished by FastPCR 6.05 (PrimerDigital, Helsinki, Finland). Primers that conformed to the criteria and summarized in Table 3 were analyzed for their complementarity with the reference sequence by Basic Local Alignment Search Tool (NCBI, Bethesda, MD, USA). The forward and reverse primer sequences were checked, and the pair that had the highest identity with the Query Sequences (reference sequences) was selected for further analysis (Table 2).

**Table 1:** ***Vibrio cholerae ompW* sequences with their GenBANK accession numbers used for primer designing**.

Strain	Accession number
*Vibrio cholerae* strain 08-5735 *ompW*gene, partial cds	FJ462446
*V. cholerae* strain 08-5739 *ompW* gene, partial cds	FJ462447
*V. cholerae* strain 08-5738 *ompW* gene, partial cds	FJ462448
*V. cholerae* strain 08-5737 *ompW* gene, partial cds	FJ462449
*V. cholerae* strain ATCC 27070 *ompW* gene, partial cds	FJ462450
*V. cholerae* strain ATCC 55056 *ompW* gene, partial cds	FJ462451
*V. cholerae* strain 08-5742 *ompW* gene, partial cds	FJ462453
*V. cholerae* O1 strain N16961 *ompW* gene, complete cds	KJ722608

**Table 2:** ***ompW* gene primers used for real-time PCR along with their properties**.

	Sequence(5′–3′)	Length (nt)	Tm (°C)	PCR_Fragment_Size (bp)	T_opt_ (°C)
Forward	Acatcagytttgaagtcctcgc	22	56.8	191	61
Reverse	Gtggtgtaattcaaacccgc	20	55.8		

**Table 3:** **Primer parameters obtained for the designed primers together with the default and ideal range as stated by Kalendar et al. (25)**.

Criteria	Default	Ideal	Obtained
Length (nt)	20–24	>21	Forward (22 nt)
			Reverse (20 nt)
T_M_ range (°C)	52–68	60–68	Forward (56.8)
			Reverse (55.8)
T_M_ 12 bases at 3′ end	30–50	41–47	Forward (42.9)
			Reverse (41.3)
CG (%)	45–65	50	Forward (47.7)
			Reverse (50.0)
Linguistic complexity (LC%)	>75	>90	Forward (95)
			Reverse (89)
Sequence quality (PQ%)	>70	>90	Forward (93)
			Reverse (87)

### Calculation of the Physical Parameters of Primers

Primer quality was calculated by the consecutive summation of the points according to the parameters: total sequence and purine–pyrimidine sequence complexity, the melting temperatures of the whole primer, and of the 12 bases from each of the terminal 3′ and 5′. The melting temperature of the 12 bases at the 3′ terminus is calculated by nearest neighbor thermodynamic parameters (26). Linguistic complexity measurements (Eqs 1–3) were performed using the alphabet-capacity *L*-gram method (27, 28). The Tm was calculated by the nearest neighbor thermodynamic parameters (26, 29). The optimal annealing temperature (*T*a) was calculated by the Eq. 4 (30).

### Real-time PCR Conditions

A Mastermix consisted of 12.5 μL 2× Power SYBR green^®^ PCR master mix containing passive reference of ROX dye (Applied Biosystems, Life Technologies, Warrington, UK), 2.5 μL of 100 nM each sense and antisense primer, 2.5 μL of DEPC treated H_2_O, and 5 μL of template DNA. The thermal conditions were maintained under the following conditions: polymerase activation at 95°C for 5 min, followed by 40 cycles of 30 s at 95°C for and 1 min at 60°C. The real-time PCR was performed using the machine Applied Biosystems StepOne™ (48-well).

### Specificity of the qPCR Assay

In order to investigate the capability of the assay to distinguish between target and non-target, DNA from 10 isolates of *E. coli*, 5 isolates of *Enterococcus* spp., 6 isolates of *Salmonella* spp., 3 isolates from *Vibrio* spp., and 7 isolates of *V. cholerae* were used as templates. The concentration of all DNA samples from the isolates was kept almost same (approximately 10 ng/μL) by diluting with DEPC-treated water or concentrating by DNA concentrator (Eppendorf Concentrator 5301).

### Melt Curve Analysis and Detection of Non-Specific Products

Four dilutions of two *V. cholerae* strains were subjected to qPCR as stated above, and the reaction mixtures containing the SYBR Green PCR products were gradually warmed to 95°C at a ramp rate of 0.3°C/s with continuous fluorescence acquisition. The melting curves were created by plotting the derivative reporter vs the temperature. The melting curve analysis was performed with duplicates of four serial dilutions of template DNA ranging from 10^6^ to 10^3^ gene copies per reaction using the ABI software version 2.2.2. The SYBR green PCR products were also resolved for identity in 1.5% agarose gel by electrophoresis.

### Sensitivity and Limits of Detection (LOD)

The DNA sample of *V. cholerae* was then serially diluted (10-fold) upto 7-log_10_ (5.46 × 10^5^ copy numbers down to 5.46 × 10^−1^) in DEPC-treated water. Five microliters from each dilution were used as template for detection. Distilled water was used as no template control.

### Calibration Standards for Standard Curves

To estimate the number of cells in a reaction, the mass of a single bacterial genomic DNA was calculated. The genome size of one *V. cholerae* was 4,033,460 bp (NCBI Genbank10952301). The molecular mass of the genome was found by multiplying the genome size with the mass of base pair. The molecular mass of *V. cholerae* was found to be 4.52 fg. The starting concentration of each stock DNA was measured by ColibriMicrovolume Spectrometer (Titertek-Berthold, Berthold Detection Systems GmbH, Bleichstrasse, Pforzheim, Germany) at absorbance 260 nm. To establish the number of cells in final reaction mixture, the stock concentration was divided by the molecular mass of the specific bacteria. The 7-log serial dilution (1:10) of the stock DNA was prepared in triplicate and the corresponding cell numbers were calculated in the final PCR reaction mixture.

### Repeatability and Reproducibility

The precision of the PCR assays was evaluated for dilutions ranging from 5.46 × 10^5^ gene copies per reaction down to 5.46 × 10^−1^ copy numbers. The dilutions were tested in four replicates in two separate PCR runs. The SD of the *C*_T_ values of each concentration was then calculated by using Eqs 1 and 2.

(1) $$\text{SD}=\sqrt{\sum \frac{{\left(C_{\text{T}}-\bar{C_{\text{T}}}\right)}^{2}}{n}}$$

where $\bar{C_{\text{T}}}$ is the mean *C*_T_ value and *n* is the number of observations. The value obtained was used to calculate the coefficient of variation, CV, with Eq. 2.

(2) $$CV=\frac{SD}{\bar{C_{\text{T}}}}$$

The intra-assay precision (repeatability) was assessed by calculating the coefficient of variation (CV%) for individual runs. The inter-assay precision (reproducibility) was calculated by determining the coefficient of variation (CV%) of both runs combined.

### Ethical Clearance

The study did not involve any human or animal related issues. Therefore, we did not seek any ethical clearance in this study. Besides, the lab is facilitated with biosafety level II functions. The test and control strains of this study fall under the BSL II category.

## Results

### Physical Parameters of Primers

The physical parameters of the primers obtained are summarized in Table 3. Sequence quality and T_M_ 12 bases at 3′ end of both forward and reverse primers, LC and length of forward primer, and CG% of reverse primer were all in the ideal range (see Table 3). All the others parameters were within the default range.

### Repeatability and Reproducibility

**Figure 1:**
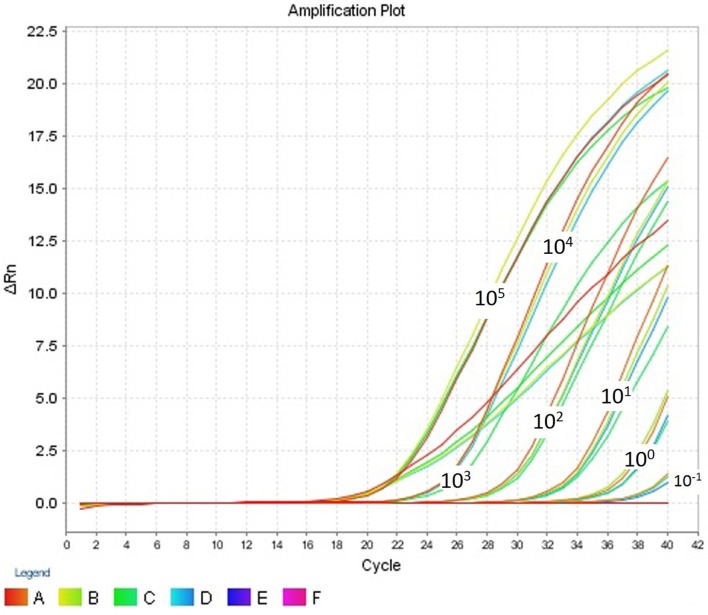
**Amplification plot (ΔRn vs Cycle) for testing the sensitivity and precision of the first replicate run**.

**Figure 2:**
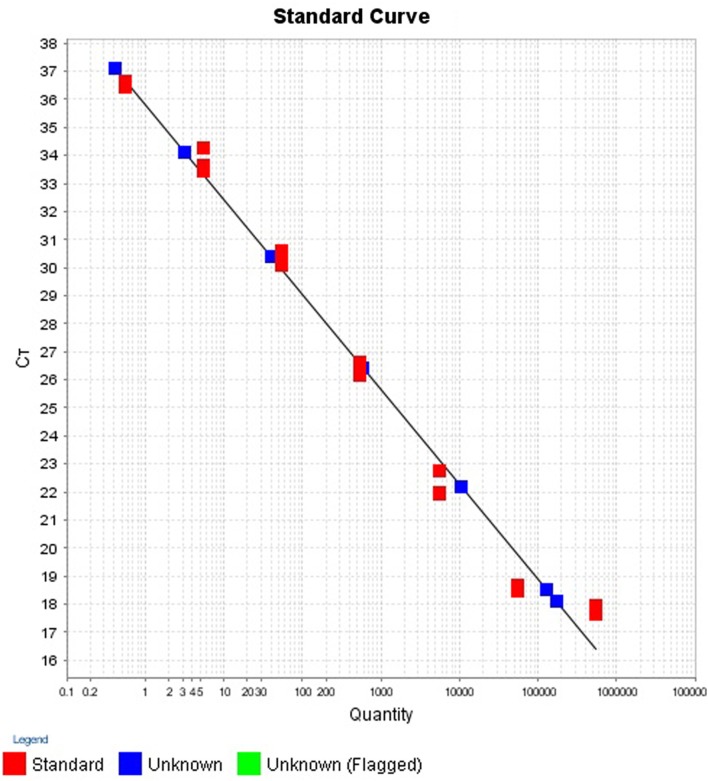
**Standard curve for the quantification of samples in first replicate run**.

**Figure 3:**
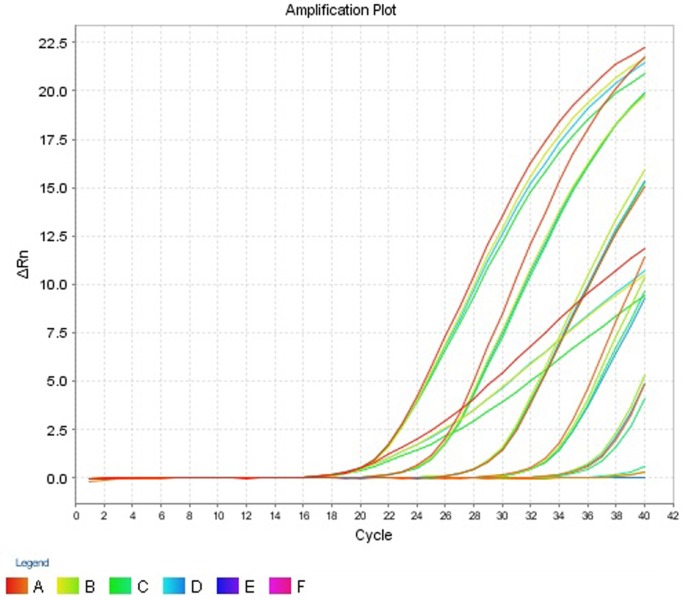
**Amplification plot (ΔRn vs Cycle) for testing the sensitivity and precision of the second replicate run**.

**Figure 4:**
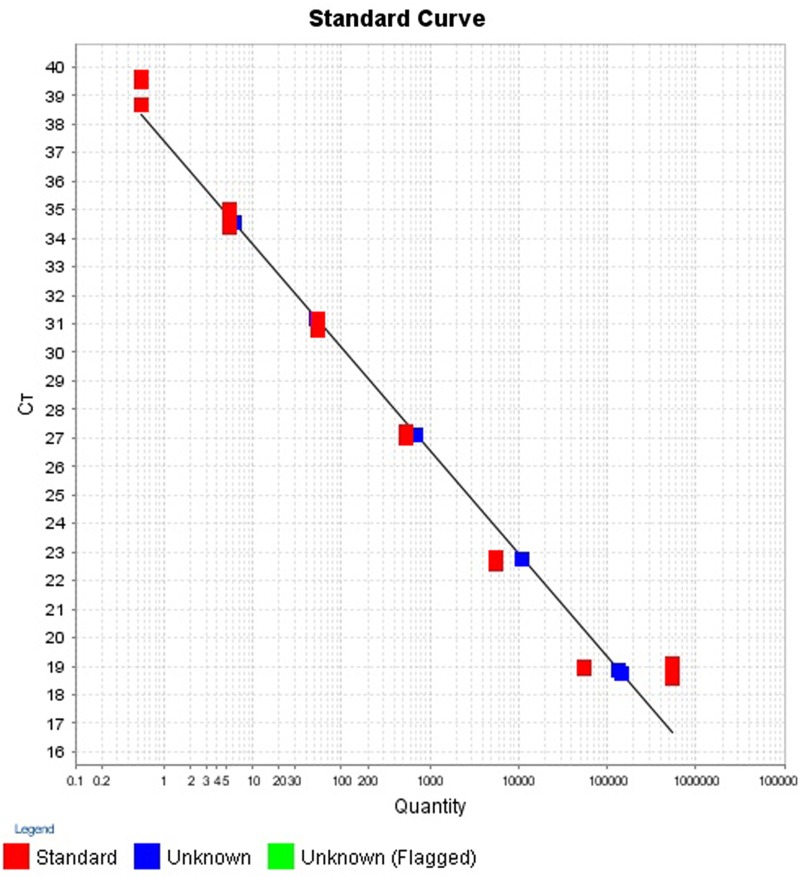
**Standard curve for the quantification of samples in second replicate run**.

The intra- and inter-run precision obtained has been summarized in Table 4. The coefficient of variation for the first replicate varied from 0.24 to 1.32 and for the second replicate the CV% ranged from 0.48 to 1.1. The CV% for the inter-run reproducibility varied from 1.08 to 3.79. The amplification plot and standard curve have been shown (Figures 1–4).

**Table 4:** **Comparison of sensitivity of detection and precision of two replicate runs**.

	Replicate run 1			Replicate run 2			
	Efficiency = 89.161%			Efficiency = 97.374%			
	Slope = −3.612			Slope = −3.386			
	*R*^2^ = 0.975			*R*^2^ = 0.982			
Copy number	SD (*n* = 4)	Mean (*n* = 4)	Coefficient of variation (CV%)	SD (*n* = 4)	Mean (*n* = 4)	Coefficient of variation (CV%)	Inter-assay CV%
5.46E10^5^	0.222951	18.806	1.185533	0.196337	17.90175	1.096748	2.838649
5.46E10^4^	0.04455	18.91	0.235588	0.089388	18.55025	0.481871	1.084382
5.46E10^3^	0.099654	22.7365	0.438301	0.382781	22.2215	1.722573	1.68125
5.46E10^2^	0.347915	27.26575	1.276015	0.190516	26.42575	0.720947	1.932086
5.46E10^1^	0.175279	31.034	0.564796	0.196538	30.3475	0.647623	1.321016
5.46E10^0^	0.28061	34.67725	0.809206	0.382505	33.864	1.129533	1.558894
5.46E10^−1^	0.517502	39.26467	1.317984	0.332131	36.731	0.904225	3.792876

### Sensitivity and LOD

The LOD or analytical sensitivity was found to be 5.46 copies since among 8 replicates. The 5.46 was the lowest gene copies that were consistently detected. For higher dilution, i.e., 0.546 copy number, the assay failed to register a *C*_T_ value in 2 of the 8 replicates.

### Specificity

**Figure 5:**
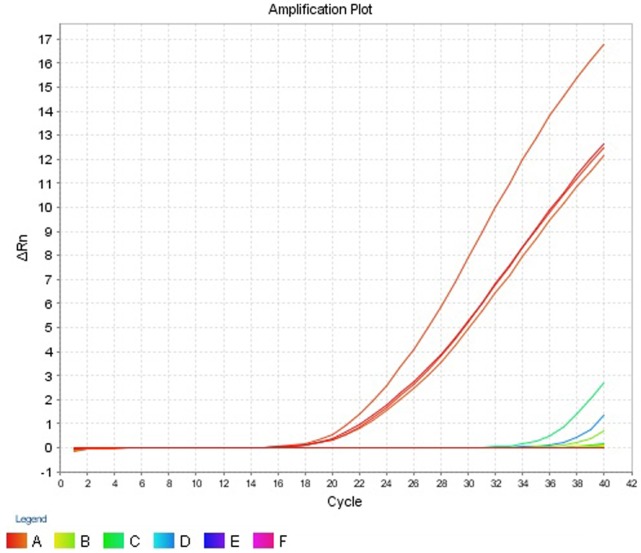
**Amplification plot (ΔRn vs Cycle) obtained for specificity test**.

The assay registered *C*_T_ values which ranged from 18.778 to 19.697 for the 4 *V. cholerae* strains and was detectable in the amplification plot (Figure 5). Two *E. coli* strains, EHEC and EIEC, had *C*_T_ values of 35.073 and 38.439, respectively. The *C*_T_ values for all other strains were undetermined. Strains which had *C*_T_ values of less than 35 were considered as *ompW* positive. Hence, the assay was able to correctly detect *V. cholerae* and gave a negative result for all other strains, thus proving the assay was *V. cholera*e specific. The results have been summarized in Table 5.

**Table 5:** **Detection of *ompW* gene for specificity test**.

Sr#.	Species	Collection or isolation number	Function of the strains	Origin	*C*_T_ value	*ompW* presence
1	*Escherichia coli* (*E. coli*)	^a^ATCC AN33859	Test strain	Clinical	U	−
2	*E. coli* EPEC	ATCC B170	Test strain	Clinical	U	−
3	*E. coli* EAEC	ATCC MG1214C2	Test strain	Clinical	U	−
4	*E. coli* ETEC	ATCC MGL-IC1	Test strain	Clinical	U	−
5	*E. coli* EHEC	NF 9422	Test strain	Clinical	U	−
6	*E. coli*	MMLA	Test strain	Clinical	U	−
7	*E. coli* EIEC	2 V	Test strain	Clinical	38.439	−
8	*E. coli* ETEC	C600	Test strain	Clinical	U	−
9	*E. coli* EIEC	H2	Test strain	Clinical	U	−
10	*E. coli* EHEC	BH29	Test strain	Clinical	35.073	−
11	*Enterococcus faecium*	T7	Test strain	Environmental	U	−
12	*E. faecium*	B10	Test strain	Environmental	U	−
13	*E. faecium*	B4	Test strain	Environmental	U	−
14	*Enterococcus faecalis*	T11	Test strain	Environmental	U	−
15	*E. faecalis*	B4PE	Test strain	Environmental	U	−
16	*Salmonella* spp.	29	Test strain	Food	U	−
17	*Salmonella* spp.	36	Test strain	Soil	U	−
18	*Salmonella* spp.	19 (b)	Test strain	Food	U	−
19	*Salmonella enteritidis*	A	Test strain	Environmental	U	−
20	*Salmonella typhimurium*	Ifo-3313	Test strain	Environmental	U	−
21	*S. typhimurium*	S1	Test strain	Environmental	U	−
22	*Vibrio parahaemolyticus*	1	Test strain	Environmental	U	−
23	*V. parahaemolyticus*	3	Test strain	Environmental	U	−
24	*Vibrio mimicus*	1	Test strain	Environmental	U	−
25	*V. cholerae* serotype O1 CT^+^	ATCC C6706	Control strain	Clinical	19.624	+
26	*V. cholerae* (*VC*) serotype O1 CT^+^	ATCC N16961	Control strain	Clinical	19.324	+
27	*VC* serotype O1 CT^−^	ATCC SA 317	Control strain	Clinical	19.697	+
28	*VC* serotype CT^+^ O139	ATCC NIHC0270	Control strain	Clinical	18.778	+
29	*V. cholerae* non-O1 CT^−^	Lab isolate-2P-16	Test strain	Environmental	22.201	+
30	*V. cholerae* non-O1 CT^−^	Lab isolate-2P-203	Test strain	Environmental	21.329	+
31	*V. cholerae* non-O1 CT^−^	Lab isolate-M-299	Test strain	Environmental	23.706	+

*^a^Reference strains: American Type Culture Collection, ATCC were collected from Laboratory of Molecular Genetics, International Centre for Diarrheal Disease Research, Bangladesh (ICDDR,B). Other isolates were obtained from clinical laboratories of ICDDR,B and Environmental Microbiology Laboratory of University of Dhaka*.

*U, undetermined*.

### Melt Curve Analysis and Detection of Non-Specific Products

**Figure 6:**
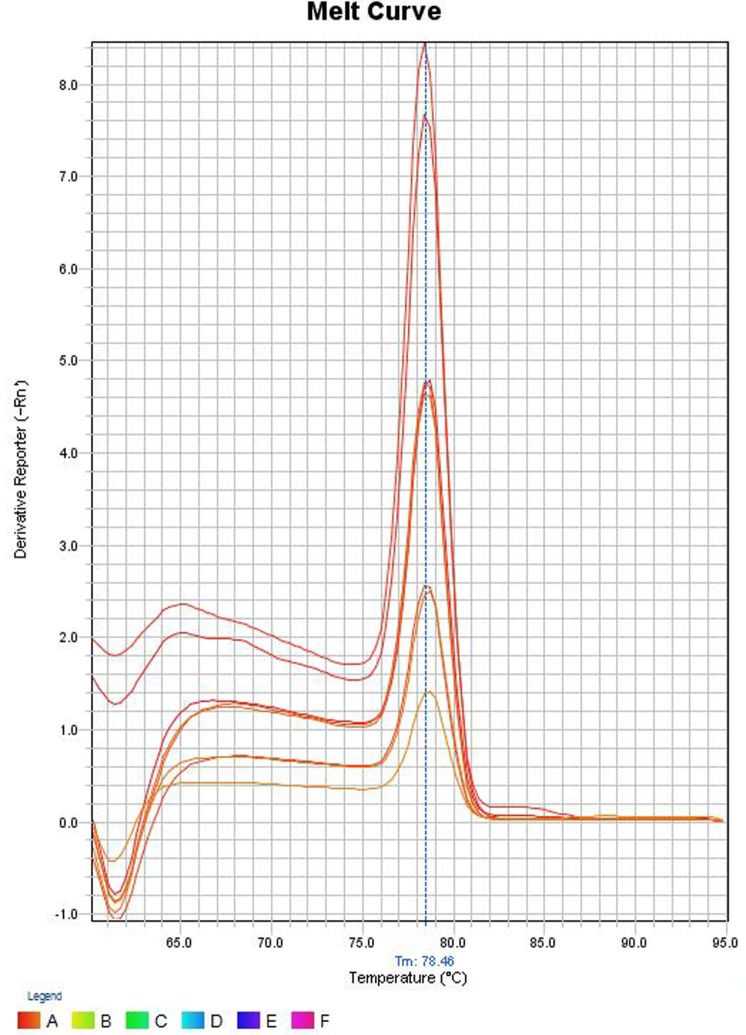
**Melt curve of SYBR green PCR products**. The *Y*-axis represents the derivative reporter (−Rn) while *x*-axis represents the temperature (°C). The figure shows a melting temperature (31) of human *ompW* PCR products as 78.46°C.

**Figure 7:**
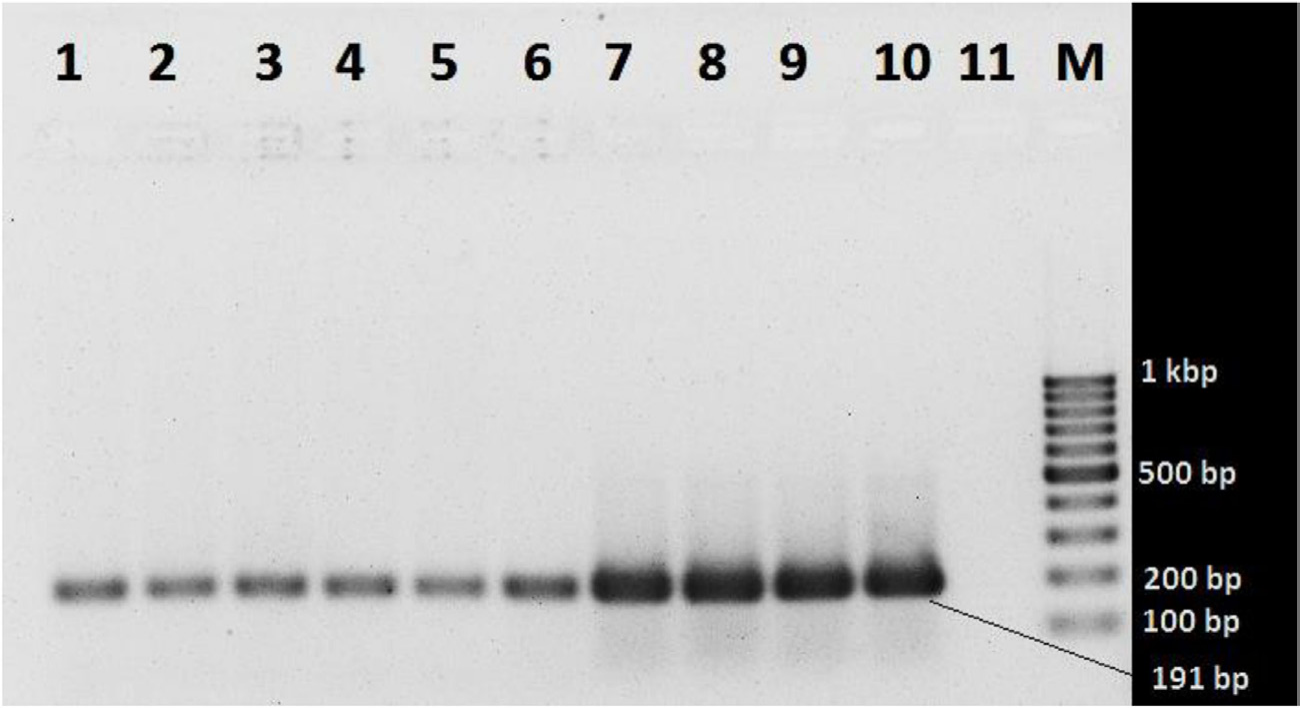
**Agarose gel electrophoresis of SYBR green PCR products**. Lanes 1 and 2 (10^3^ gene copy): *Vibrio cholerae* O1 ATCC N16961 and *V. cholerae* O139 ATCC NIHC0270, respectively; lanes 3 and 4 (10^4^ copies): *V. cholerae* O1 ATCC N16961 and *V. cholerae* O139 NIHC0270 ATCC, respectively; lanes 5 and 6 (10^5^ copies): *V. cholerae* O1 ATCC N16961 and *V. cholerae* O139NIHC0270ATCC, respectively; lanes 7 and 8 (10^6^ copies): *V. cholerae* O1 ATCC N16961 and *V. cholerae* O139NIHC0270ATCC, respectively; lanes 9 and 10 (10^7^ copies): *V. cholerae* O1 ATCC N16961 and *V. cholerae* O139NIHC0270ATCC, respectively; Lane 10 (M): molecular weight marker (100 bp DNA Ladder, Karl Roth, Germany), 11 no template control.

In the melt curve (Figure 6), a single distinct peak was seen, indicating that all the PCR products had similar Tm values which was approximately 78.46°C. Agarose gel electrophoresis of SYBR green PCR products gave a single distinct band of about 191 bp (Figure 7). It could be concluded that neither secondary non-specific products nor primer dimers were formed.

## Discussion

We have developed a real-time assay with designed primers for the detection and quantification of *V. cholerae*. The assay was based on SYBR Green PCR Mastermix and targeted the *ompW* gene, which is present in all species of *V. cholerae*. Initially, the physical properties of primers were assessed, followed by validation of sensitivity, precision, specificity, and melt curve analysis.

The LC describes nucleotide arrangement and composition of a sequence and the likelihood of PCR success of each primer. LC values of 80 and higher serve as excellent candidate primers. The primers developed had LC values of 89 and 95 for reverse and forward primers, respectively. Low-complexity regions such as Simple Sequence Repeats, imperfect direct or inverted repeats, triple-stranded DNA structures, and G/C quadruplexes (32) were unlikely to be formed if primers with high LC values are used. The parameter “Primer Quality” determines the possibility of primer dimer formation since dimers reduces the PQ value. The designed primers had PQ values of 87 and 93 for reverse and forward primers, respectively. Thus, these high values suggest that self-complementarity was not apparent. Two terminal C/G bases, recommended for increased PCR efficiency (33) were present in the designed primers.

The efficiency of a PCR assay is the amount of DNA that is amplified in each cycle. An efficiency of 100% indicates the target DNA has been doubled. The efficiencies obtained for the replicates 1 and 2 were 89.16 and 97.37%, respectively. Generally, efficiencies ranging from 90 to 100% are considered to be satisfactory. Inadequate primer design, production of non-specific amplicons and primer dimers may be responsible for reduced efficiencies (34). This is, however, only an estimate of the PCR efficiency and a real test sample, such as food, may contain inhibitory substances that decrease the PCR efficiency (35).

The precision of the assay was assessed by calculating both repeatability (intra-assay precision) and reproducibility (inter-assay precision). The coefficient of variation (CV%) for the repeatability ranged from 0.24 to 1.32 for both replicates. The CV% for the reproducibility varied from 1.08 to 3.79. The reproducibility is an important parameter since changed conditions such as different equipment and operators might affect the outcome. Pipetting and other human errors might account for poor precision. The precision usually increases with decreasing gene copy concentration (34) but this pattern was not observed for the developed assay. Retesting is required if the % CV of the PCR replicates exceeded 30% (36). All the CV% values for the assay were acceptable.

Specificity is ability to detecting chosen gene in the presence of non-specific DNA (34). The specificity is an important parameter since, in clinical and food samples, DNA from a wide range of organisms might be present. The developed assay was able to correctly detect the 7 *V. cholerae* and gave *C*_T_ values that ranged from 18.778 to 23.706. Though the assay did not give any *C*_T_ values for the 22 non-*V. cholerae* strains (Table 5), two *E. coli* strains—*E. coli* EIEC 2V, *E. coli* EHEC BH29 showed *C*_T_ values of 38.439 and 35.073 respectively. Since the cut point *C*_T_ value for ABI StepOne real-time machine is between >8 and <35, these *C*_T_ values of *E. coli* strains can be considered as negative results.

The LOD is the lowest gene copy number that the assay is able to consistently detect (37). A satisfactory LOD is 10 gene copies per reaction, and the assay was able to meet this requirement by consistently detecting 5.46 copies of the gene. The LOD sheds light on how sensitive the assay is.

The assay was evaluated for its ability to detect *V. cholerae* O1/O139 and non-O1/non-O139 in food and environmental samples over different dilutions. It was observed that drinking water, pond water, shrimp, and boiled rice spiked with these strains registered *C*_T_ values that ranged from 16.33 to 26.78 (Table 6).

**Table 6:** **Evaluation of the assay using direct environmental samples**.

#SL	Strain	Dilution	*C*_T_	Sample type
1	*Vibrio cholerae* CT^+^ O139	10^5^	16.88	Spiked drinking water
2	*V. cholerae* CT^+^ O1	10^5^	16.87	Spiked drinking water
3	*V. cholerae* CT^+^ O1	10^4^	20.18	Spiked drinking water
4	*V. cholerae* CT^+^ O1	10^3^	24.67	Spiked drinking water
5	*V. cholerae* CT^−^ non-O1/non-O139	10^4^	20.15	Spiked drinking water
6	*V. cholerae* CT^−^ non-O1/non-O139	10^3^	23.97	Spiked drinking water
7	–	–	U	Unspiked drinking water
8	–	–	U	Unspiked drinking water
9	*V. cholerae* CT^+^ O139	10^5^	16.84	Spiked pond water
10	*V. cholerae* CT^+^ O1	10^5^	16.84	Spiked pond water
11	*V. cholerae* CT^+^ O1	10^4^	20.85	Spiked pond water
12	*V. cholerae* CT^+^ O1	10^3^	26.81	Spiked pond water
13	*V. cholerae* CT^−^ non-O1/non-O139	10^4^	20.29	Spiked pond water
14	*V. cholerae* CT^−^ non-O1/non-O139	10^3^	24.83	Spiked pond water
15	–	–	U	Unspiked pond water
16	–	–	U	Unspiked pond water
17	*V. cholerae* CT^+^ O139	10^5^	16.75	Spiked boiled rice
18	*V. cholerae* CT^+^ O1	10^5^	16.37	Spiked boiled rice
19	*V. cholerae* CT^+^ O1	10^4^	20.67	Spiked boiled rice
20	*V. cholerae* CT^+^ O1	10^3^	24.19	Spiked boiled rice
21	*V. cholerae* CT^−^ non-O1/non-O139	10^4^	20.34	Spiked boiled rice
22	*V. cholerae* CT^−^ non-O1/non-O139	10^3^	26.78	Spiked boiled rice
23	–	–	U	Unspiked boiled rice
24	–	–	U	Unspiked boiled rice
25	*V. cholerae* CT^+^ O139	10^5^	16.75	Spiked shrimp
26	*V. cholerae* CT^+^ O1	10^5^	16.33	Spiked shrimp
27	*V. cholerae* CT^+^ O1	10^4^	21.00	Spiked shrimp
28	*V. cholerae* CT^+^ O1	10^3^	23.97	Spiked shrimp
29	*V. cholerae* CT^−^ non-O1/non-O139	10^4^	20.37	Spiked shrimp
30	*V. cholerae* CT^−^ non-O1/non-O139	10^3^	25.36	Spiked shrimp
31	–	–	U	Unspiked shrimp
32	–	–	U	Unspiked shrimp
33	–	–	U	No template control
34	*V. cholerae* CT^+^ O1	10^6^	10.13	Positive control

To assess if the assay is affected by interference from non-target DNA, unspiked drinking water, pond water, shrimp, and boiled rice were examined by qPCR. Before this assessment, absence of *V. cholerae* was confirmed. Results showed that no *C*_T_ values were obtained for these unspiked food and water samples. Thus, this assay is suitable for detecting both *V. cholerae* O1/O139 and non-O1/non-O139 in food and environmental samples since non-specific amplification was not seen in negative controls.

Melt curve analysis was done to assess whether secondary products such as primer dimers or non-specific products were formed. The melt curve gave a single peak with a Tm value of about 78.46°C. Agarose gel electrophoresis of SYBR Green PCR products gave a single band at 191 bp. These results suggest that the amplification was specific and only one type of amplicon was produced. Non-specific products hamper the efficiency of the assay and affect precision. Non-specific products were absent suggests that the primer design was adequate. The primers were specific and intended amplicons were produced. We can conclude the primers were not complementary to one another since primer dimers were not produced.

Many assays have been developed for detection and quantification of *V. cholerae* (19–24). Though impressive none of these presented any statistical figures (such as coefficient of variation) which would inform us about the reproducibility and repeatability. Many of these assays did not undergo melt curve analysis or the PCR products were not subjected to agarose gel electrophoresis and hence we do not know their status regard the formation of non-specific products. Since they are multiplex in nature, they add to the cost and hence are not suitable for purposes. For instance, during quality control testing of seafood where only quantification is required to see if the levels in food is acceptable to the standards set by the governing bodies.

An extremely impressive multiplex real-time assay has been developed by Bliem and colleagues (38). The assay is multiplex in nature, and hence the use of multiple primers might add to the cost. The assay developed by Bliem and colleagues had inter-assay variance of 2–28% for *ompW*. But our assay, which utilizes a primer for *ompW* gene with different sequence, was more precise with inter-assay variance of 1.08–3.79.

Future objectives of our study might include the optimization of this assay to detect and quantify *V. cholerae* in food, water, and clinical samples. Some samples might contain inhibitory substances that decrease PCR efficiency (35) and hence optimization of the methods involving sample processing, DNA extraction, and assay itself might be required.

## Author Contributions

PJ and AB are the principal investigators of the project and contributed to the manuscript revision and final version approval to be published. RR conducted the study in the laboratory, performed statistical analysis, and wrote the first draft of the manuscript. ST contributed to revising the manuscript critically for important intellectual content. JF contributed to the study designing, implementation, manuscript reviewing, and revising it critically. The authors have agreed to be accountable for answering questions related to the accuracy and integrity of the work appropriately done.

## Conflict of Interest Statement

The authors declare that the research was conducted in the absence of any commercial or financial relationships that could be construed as a potential conflict of interest.
